# Novel Application of Point-of-Care Ocular Ultrasound in a Left Central Retinal Artery Occlusion

**DOI:** 10.7759/cureus.7518

**Published:** 2020-04-02

**Authors:** Rachel E Bridwell, Jesse Wray, Joshua Oliver, Amber Cibrario, Melissa Myers

**Affiliations:** 1 Emergency Medicine, Brooke Army Medical Center, Fort Sam Houston, USA

**Keywords:** central retinal artery occlusion, ultrasound

## Abstract

Central retinal artery occlusion represents a vision-threatening entity in those presenting with monocular painless vision loss, especially in the elderly and those with cardiovascular comorbidities. While confirmation of this diagnosis requires consultation with an ophthalmologist, prompt recognition is the crucial action of the emergency physician to help reverse retinal ischemia and save vision. Here we describe the case of a central retinal artery occlusion identified on point-of-care ocular ultrasound and confirmed by fluorescein angiography.

## Introduction

Central retinal artery occlusion (CRAO) is a rare but vision-threatening event occurring in 1-2 in 100,000 patients, though this increases with age, occurring 10 times more commonly in patients over 80 years of age [1-3]. Direct fundoscopy, a mainstay of initial CRAO diagnosis until fluorescein angiography can be performed, is not a central skill for the emergency physician, which is the mainstay of initial CRAO diagnosis. There is a paucity of evidence in the literature in which point-of-care ultrasound (POCUS) has been used to diagnose CRAO. We present the case below with painless monocular vision loss of a thrombus identified on emergency POCUS.

## Case presentation

A 63-year-old woman with a history of coronary artery disease, diabetes mellitus, and atrial fibrillation not on anticoagulation presented to the emergency department (ED) with acute-onset persistent painless vision loss in her left eye two hours prior to arrival. Review of systems was notable only for a left-sided temporal headache.

**Figure 1:**
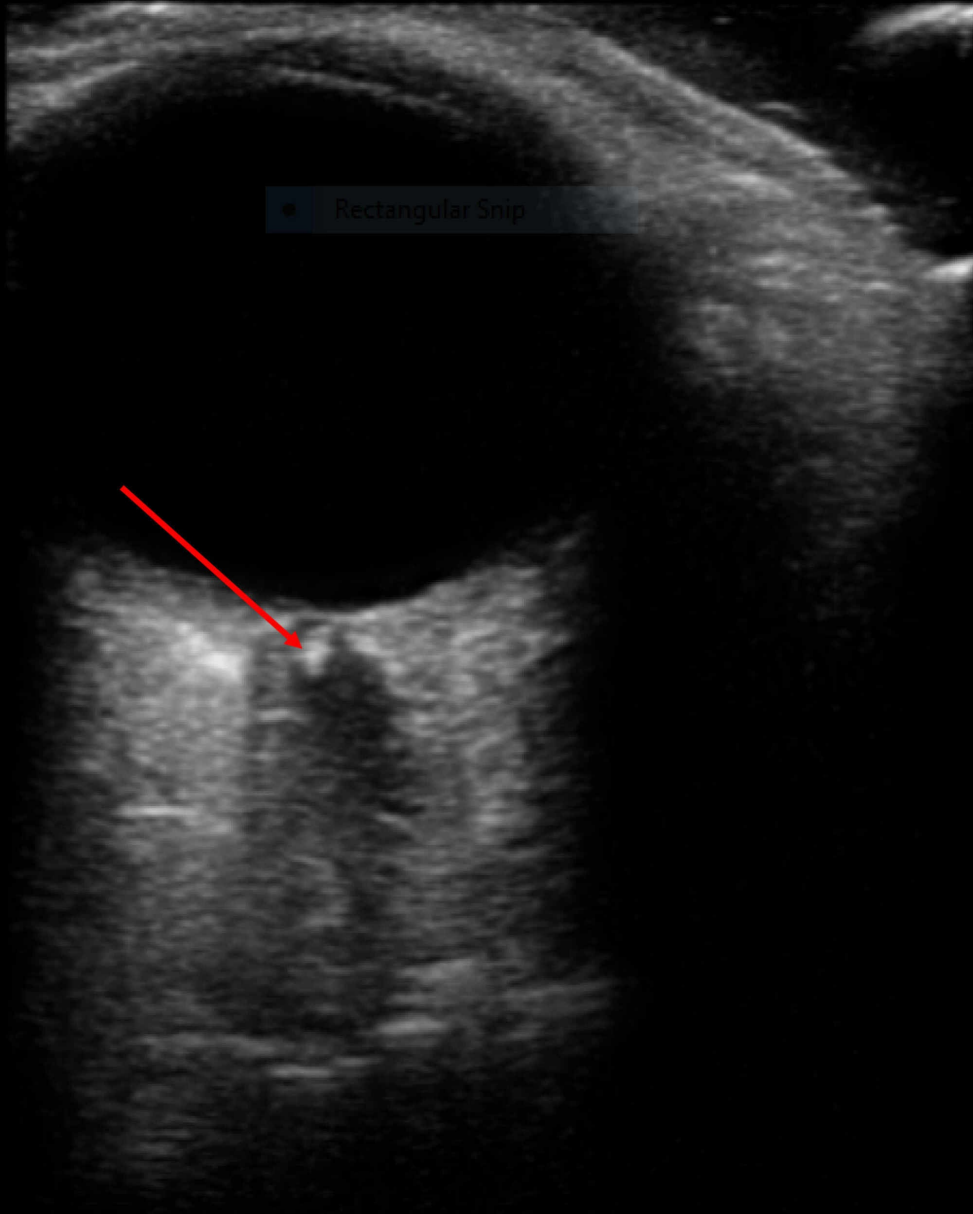
Transverse linear array ultrasound of the left eye demonstrating central retinal artery occlusion (red arrow).

**Figure 2:**
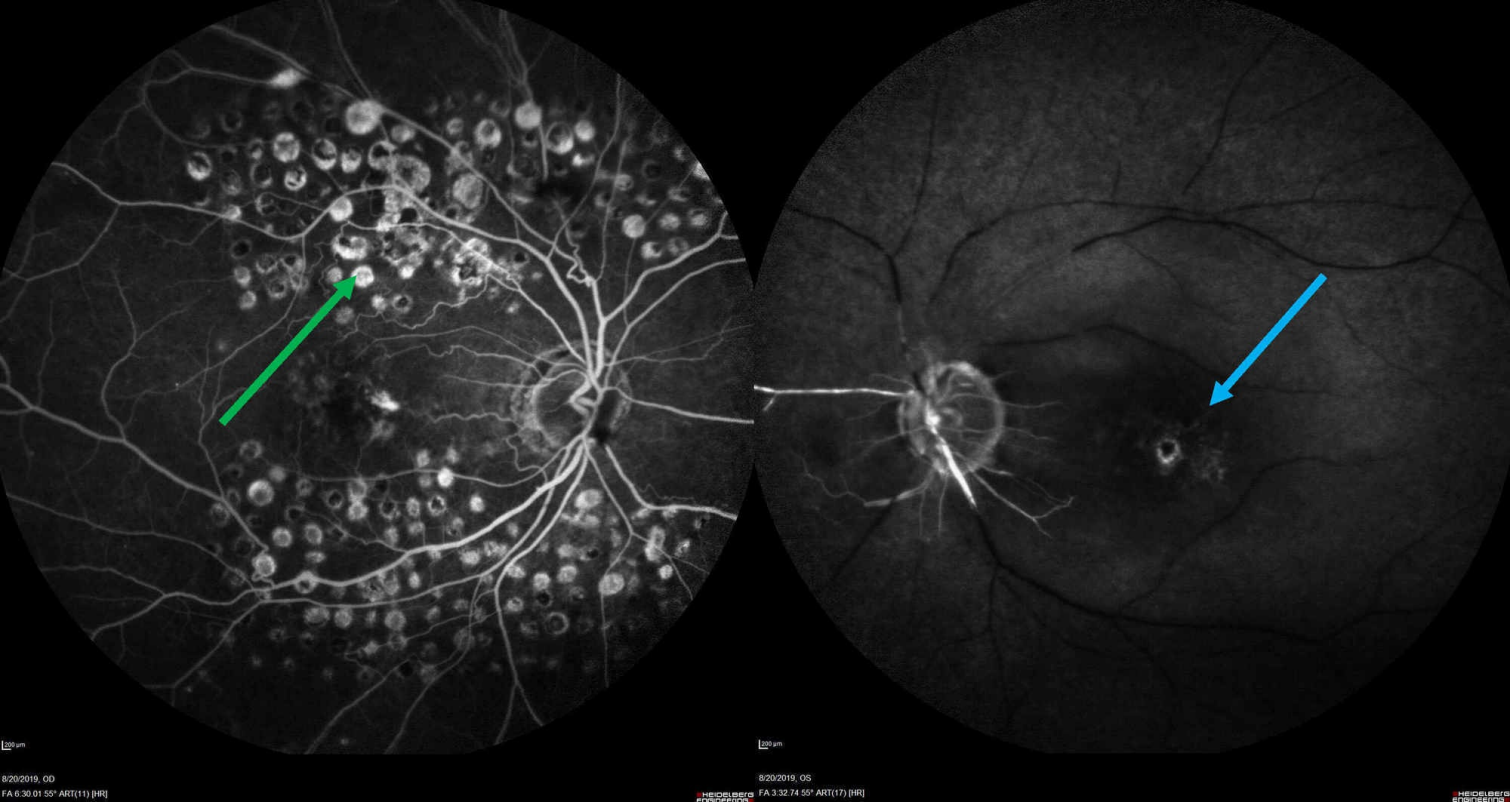
Fluorescein angiography demonstrating central retinal artery occlusion without vascular flow in the left eye (blue arrow). Additional demonstration of argon laser scarring from retinal photocoagulation (green arrow)

On arrival, the vital signs were within normal limits. The physical examination was notable for an inability to perceive light or movement in the left eye with an afferent pupillary defect. POCUS demonstrated a hyperechoic circular structure in the center of the left optic nerve (Figure 1). A computed tomography (CT) of the head and CT angiography of the neck were obtained, which demonstrated atherosclerotic disease of the bilateral carotid, temporal, and ophthalmic arteries, but there was no evidence of acute intracranial ischemia or hemorrhage. She was placed on 100% fraction of inspired oxygen due to a concern for CRAO. Both neurology and ophthalmology providers were consulted, who recommended hyperbaric oxygen therapy. Because giant cell arteritis (GCA) was an alternative possible etiology of the presentation, she was empirically started on high-dose corticosteroids. A subsequent temporal artery biopsy found no evidence of vasculitis. She was admitted to the medical ward and underwent a hyperbaric oxygen protocol without improvement in her visual deficit. CRAO was observed on direct fundoscopy and confirmed on fluorescein angiography (Figure 2). Additional cerebrovascular accident (CVA) workup to include transthoracic echocardiography and magnetic resonance imaging (MRI) with angiography demonstrated no patent foramen ovale or acute findings. After discharge, an outpatient ophthalmology follow-up demonstrated no light perception in the left eye.

## Discussion

CRAO represents a portion of patients presenting with monocular painless vision loss, in which retinal ischemic time determines vision. While rare, its incidence increases exponentially until the eighth decade of life and is associated with coronary artery disease, diabetes mellitus, CVA, transient ischemic attack, hyperlipidemia, smoking, and hypercoagulable states, with embolism representing as the most common etiology [2,3]. Currently, the mainstay of diagnostic workup in the ED relies heavily on direct fundoscopy, which is a dying art for the emergency physician. Traditional diagnostic criteria include the presence of “box-carring” of the retinal vessels in conjunction with retinal opacity with a cherry red spot [2]. After establishing these examination findings, the diagnosis is traditionally confirmed with a fluorescein angiography [1]. There are only a few limited studies describing the use of ultrasound to identify CRAO [4,5]. There are no definitive sensitivities for POCUS, though 31% of CRAOs demonstrated retrobulbar hyperechoic foci on orbital color Doppler imaging, suggesting the possible utility of ultrasound [4]. The presence of a visible thrombus such as the one described may expedite the workup; primate models and human observational studies have demonstrated that the duration of retinal ischemic time significantly determines visual damage [1,6,7].

In ED management, any clinical presentation suggesting GCA should initiate inflammatory markers and prompt administration of steroids. In a study of retinal artery occlusions, 12 cases experienced CRAO secondary to GCA [2]. In this small subset, GCA causes posterior ciliary artery and cilioretinal artery occlusion [8]. CRAO also portends a higher risk of subsequent myocardial infarction or cerebral infarction, especially within the first week following a CRAO [1]. Emergency physicians should not only recognize the vision-threatening aspect of this disease but also recognize it as a marker of poor cardiovascular status with an increased risk of other cardiovascular morbidity and mortality [9,10].

Workup in the ED should include a parallel CVA workup with an MRI with diffusion-weighted imaging if possible, as CRAO is a stroke equivalent with retinal tissue ischemia [11]. The treatment of CRAO presents the ultimate challenge as a few successful treatments have been described in data. Ocular massage, laser embolectomy, minimization of intra-ocular pressure, hyperbaric oxygen, and intra-arterial thrombolysis have not demonstrated improvement in vision [12-16]. Given the high risk of both cardiovascular complications and additional hypercoagulability workup, patients should be admitted [2].

## Conclusions

CRAO is a rare but vision-threatening diagnosis in those presenting with painless monocular vision loss, more common in the elderly and those with cardiovascular medical comorbidities. Prompt recognition and diagnosis is paramount in reversing the ongoing retinal vascular ischemia. Previously described in only a few cases, POCUS may identify distal thrombi and expedite this time-sensitive workup.
